# 5-Amino-3-methyl-1,2-oxazole-4-carbonitrile

**DOI:** 10.1107/S1600536812032667

**Published:** 2012-07-25

**Authors:** Samad Shoghpour, Amir Keykha, Hadi Amiri Rudbari, Mohammad Rahimizadeh, Mehdi Bakavoli, Mehrdad Pourayoubi, Francesco Nicolò

**Affiliations:** a Department of Chemistry, Ferdowsi University of Mashhad, Mashhad 91779, Iran; b Dipartimento di Chimica Inorganica, Vill. S. Agata, Salita Sperone 31, Università di Messina, 98166 Messina, Italy

## Abstract

In the title compound, C_5_H_5_N_3_O, the isoxazole ring is essentially planar, with a maximum deviation of 0.007 (1) Å from the least-squares plane. The N atom of the amine group exhibits *sp* ^2^ character (sum of bond angles around this atom = 358°). In the crystal, mol­ecules are aggregated by two kinds of N—H⋯N hydrogen bonds into fused *R*_2_^2^(12) and *R*_6_^6^(26) rings, forming a slightly puckered two-dimensional array lying parallel to (101).

## Related literature

For the biological activities of isoxazole derivatives, see: Mantegani *et al.* (2011 ▶); Ali *et al.* (2011 ▶); Panda *et al.* (2009 ▶); Özdemir *et al.* (2007 ▶); Banerjee *et al.* (1994 ▶); Makoto *et al.* (2011 ▶). For background to push–pull nitriles, see: Ziao *et al.* (2001 ▶); Hao *et al.* (2005 ▶). For hydrogen-bond motif definitions, see: Bernstein *et al.* (1995 ▶).
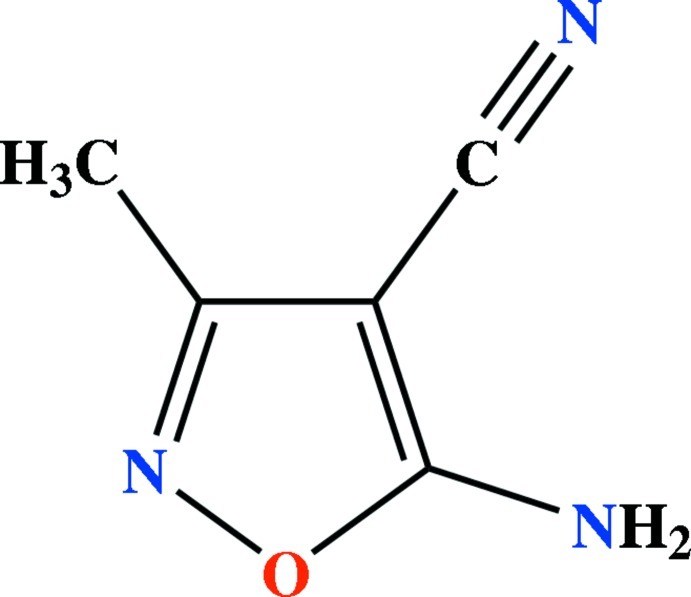


## Experimental

### 

#### Crystal data

C_5_H_5_N_3_O
*M* *_r_* = 123.12
Monoclinic, 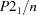

*a* = 3.8779 (2) Å
*b* = 18.8518 (11) Å
*c* = 8.2015 (4) Å
β = 100.780 (2)°
*V* = 588.99 (5) Å^3^
*Z* = 4
Mo *K*α radiation
μ = 0.10 mm^−1^
*T* = 296 K
0.56 × 0.26 × 0.20 mm

#### Data collection

Bruker APEXII CCD diffractometer
Absorption correction: multi-scan (*SADABS*; Bruker, 2008 ▶) *T*_min_ = 0.674, *T*_max_ = 0.745
5275 measured reflections
1277 independent reflections
1072 reflections with *I* > 2σ(*I*)
*R*_int_ = 0.020

#### Refinement

*R*[*F* ^2^ > 2σ(*F* ^2^)] = 0.035
*wR*(*F* ^2^) = 0.107
*S* = 1.07
1277 reflections
91 parameters
H atoms treated by a mixture of independent and constrained refinement
Δρ_max_ = 0.19 e Å^−3^
Δρ_min_ = −0.16 e Å^−3^

### 

Data collection: *APEX2* (Bruker, 2007 ▶); cell refinement: *SAINT* (Bruker, 2007 ▶); data reduction: *SAINT*; program(s) used to solve structure: *SHELXS97* (Sheldrick, 2008 ▶); program(s) used to refine structure: *SHELXL97* (Sheldrick, 2008 ▶); molecular graphics: XPW (Siemens, 1996 ▶); software used to prepare material for publication: *SHELXTL* (Sheldrick, 2008 ▶) and *enCIFer* (Allen *et al.*, 2004 ▶).

## Supplementary Material

Crystal structure: contains datablock(s) I, global. DOI: 10.1107/S1600536812032667/gk2513sup1.cif

Structure factors: contains datablock(s) I. DOI: 10.1107/S1600536812032667/gk2513Isup2.hkl

Supplementary material file. DOI: 10.1107/S1600536812032667/gk2513Isup3.cml

Additional supplementary materials:  crystallographic information; 3D view; checkCIF report

## Figures and Tables

**Table 1:** Hydrogen-bond geometry (Å, °)

*D*—H⋯*A*	*D*—H	H⋯*A*	*D*⋯*A*	*D*—H⋯*A*
N2—H2*A*⋯N1^i^	0.842 (19)	2.118 (19)	2.9567 (16)	174.2 (16)
N2—H2*B*⋯N3^ii^	0.870 (18)	2.174 (18)	3.0402 (17)	173.8 (15)

Symmetry codes: (i) 
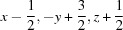
; (ii) 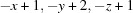.

**Fig. 1.**
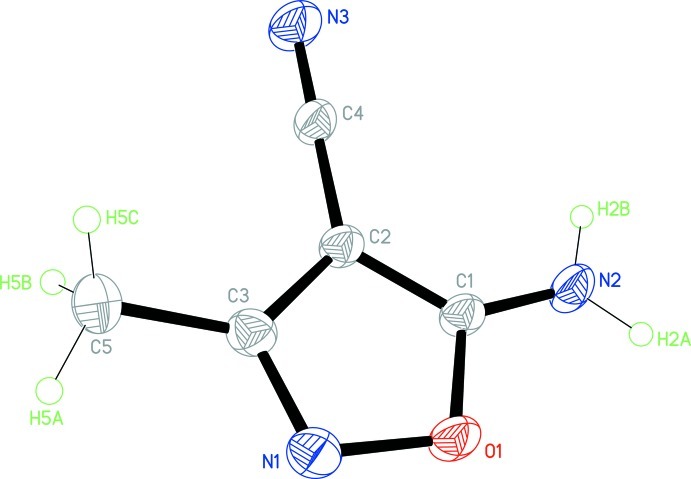
The molecular structure of title compound, showing the atom-numbering scheme and displacement ellipsoids at the 50% probability level.

**Fig. 2.**
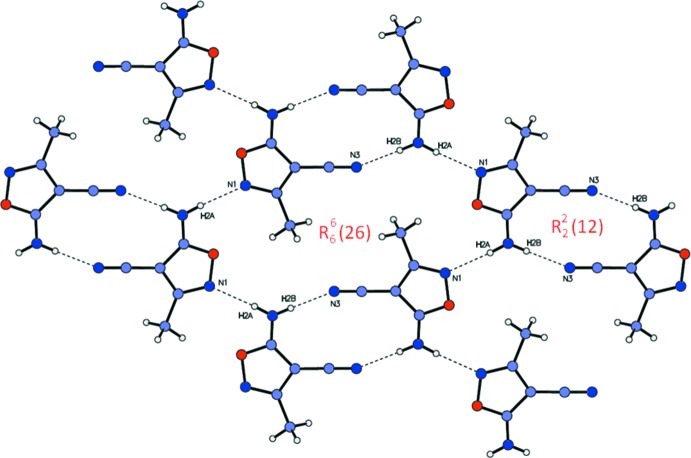
A view of two-dimensional structure in the title compound showing the R22(12) and R66(26) graph-set motifs, built from N—H···N hydrogen bonds (dashed lines).

## supplementary crystallographic information

### Comment

Isoxazoles are an important class of heterocyclic compounds which are widely used in medicinal chemistry. A number of isoxazole derivatives are known to act as anti-tumor (Mantegani *et al.*, 2011), anti-HIV (Ali *et al.*, 2011), anti-inflammatory, antibacterial (Panda *et al.*, 2009), antidepressant, anticonvulsant (Özdemir *et al.*, 2007) and anthelmintic (Banerjee *et al.*, 1994) agents. Isoxazole derivatives are also utilized in therapy in the treatment of diabetes, obesity or hyperlipemia (Makoto *et al.*, 2011). Considering the potential of the title compound as a pharmaceutical intermediate, its crystal structure is reported here.

The title molecule (Fig. 1) exhibits a planar isoxazole ring with a maximum deviation of 0.007 (1) Å for atom C2. The sum of the bond angles around the N atom of the amine group (358°) is in accordance with *sp*^2^ hybridization.

As has been described for related push–pull nitriles (Ziao *et al.*, 2001; Hao *et al.*, 2005), there is a conjugative interaction between the amino nitrogen lone pair and the nitrile nitrogen *via* the C1═C2 bond which increases the hydrogen-bonding acceptor capability of the nitrile nitrogen. Thus, the amino nitrogen lone pair is not available for a hydrogen-bond interaction and it does not form any hydrogen bond as an acceptor. In addition, the C4≡N3 bond length [1.1424 (16) Å] is typical of the nitrile bond lengths found in push–pull nitriles.

In the crystal structure (Fig. 2), molecules are linked by N—H···N_cyano_ (N2···N3 = 3.0402 (17) Å) and N—H···N_isoxazole_ (N2···N1 = 2.9567 (16) Å) hydrogen bonds (Table 1) building *R*_2_^2^(12) and *R*_6_^6^(26) rings (Bernstein *et al.*, 1995) in a two-dimensional arrangement along the (101) plane.

### Experimental

To a solution of hydroxylamine hydrochloride (13.9 g, 0.2 mol) in 10% sodium hydroxide (80 ml), (1-ethoxyethylidene)malononitrile (27.23 g, 0.2 mol) was added dropwise at 323 K under vigorous stirring condition. The temperature was kept below 323 K by making this addition slowly and by addition of small amount of ice. After stirring for an additional 1.5 h at approximately 293 K, the resulting solid was filtered and washed with water. Single crystals of title compound were obtained from a solution of aqueous ethanol after slow evaporation at room temperature.

### Refinement

All H atoms were located on a final ΔF map. The positions of H atoms from the methyl group were determined geometrically (C—H = 0.96 Å) and these atoms were refined as riding with *U*_iso_(H) = 1.5*U*_eq_(C). The H atoms of the amine group were freely refined.

### Crystal data

**Table:**

C_5_H_5_N_3_O	*F*(000) = 256
*M**_r_* = 123.12	*D*_x_ = 1.388 Mg m^−^^3^
Monoclinic, *P*2_1_/*n*	Mo *K*α radiation, λ = 0.71073 Å
Hall symbol: -P 2yn	Cell parameters from 2187 reflections
*a* = 3.8779 (2) Å	θ = 2.8–26.2°
*b* = 18.8518 (11) Å	µ = 0.10 mm^−^^1^
*c* = 8.2015 (4) Å	*T* = 296 K
β = 100.780 (2)°	Irregular, colourless
*V* = 588.99 (5) Å^3^	0.56 × 0.26 × 0.20 mm
*Z* = 4	

### Data collection

**Table:**

Bruker APEXII CCD diffractometer	1277 independent reflections
Radiation source: fine-focus sealed tube	1072 reflections with *I* > 2σ(*I*)
Graphite monochromator	*R*_int_ = 0.020
φ and ω scans	θ_max_ = 27.0°, θ_min_ = 3.3°
Absorption correction: multi-scan (*SADABS*; Bruker, 2008)	*h* = −4→4
*T*_min_ = 0.674, *T*_max_ = 0.745	*k* = −24→23
5275 measured reflections	*l* = −10→10

### Refinement

**Table:**

Refinement on *F*^2^	Primary atom site location: structure-invariant direct methods
Least-squares matrix: full	Secondary atom site location: difference Fourier map
*R*[*F*^2^ > 2σ(*F*^2^)] = 0.035	Hydrogen site location: inferred from neighbouring sites
*wR*(*F*^2^) = 0.107	H atoms treated by a mixture of independent and constrained refinement
*S* = 1.07	*w* = 1/[σ^2^(*F*_o_^2^) + (0.0631*P*)^2^ + 0.0596*P*] where *P* = (*F*_o_^2^ + 2*F*_c_^2^)/3
1277 reflections	(Δ/σ)_max_ = 0.001
91 parameters	Δρ_max_ = 0.19 e Å^−^^3^
0 restraints	Δρ_min_ = −0.16 e Å^−^^3^

### Special details

**Table:**

Geometry. All s.u.'s (except the s.u. in the dihedral angle between two l.s. planes) are estimated using the full covariance matrix. The cell s.u.'s are taken into account individually in the estimation of s.u.'s in distances, angles and torsion angles; correlations between s.u.'s in cell parameters are only used when they are defined by crystal symmetry. An approximate (isotropic) treatment of cell s.u.'s is used for estimating s.u.'s involving l.s. planes.
Refinement. Refinement of *F*^2^ against ALL reflections. The weighted *R*-factor *wR* and goodness of fit *S* are based on *F*^2^, conventional *R*-factors *R* are based on *F*, with *F* set to zero for negative *F*^2^. The threshold expression of *F*^2^ > 2σ(*F*^2^) is used only for calculating *R*-factors(gt) *etc*. and is not relevant to the choice of reflections for refinement. *R*-factors based on *F*^2^ are statistically about twice as large as those based on *F*, and *R*-factors based on ALL data will be even larger.

### Fractional atomic coordinates and isotropic or equivalent isotropic displacement parameters (Å^2^)

**Table:**

	*x*	*y*	*z*	*U*_iso_*/*U*_eq_	
O1	0.8940 (2)	0.77984 (4)	0.37710 (11)	0.0430 (3)	
C2	0.8212 (3)	0.89315 (6)	0.30725 (13)	0.0343 (3)	
N1	0.9900 (3)	0.78822 (6)	0.21727 (13)	0.0454 (3)	
N2	0.6888 (4)	0.84546 (6)	0.57048 (14)	0.0463 (3)	
N3	0.6449 (4)	1.02356 (6)	0.30830 (16)	0.0577 (4)	
C4	0.7269 (3)	0.96534 (6)	0.30883 (14)	0.0384 (3)	
C3	0.9420 (3)	0.85487 (7)	0.17972 (14)	0.0381 (3)	
C5	1.0051 (4)	0.88260 (8)	0.01801 (16)	0.0518 (4)	
H5A	1.1058	0.8459	−0.0394	0.078*	
H5B	1.1637	0.9221	0.0372	0.078*	
H5C	0.7866	0.8977	−0.0482	0.078*	
C1	0.7919 (3)	0.84280 (6)	0.42663 (14)	0.0347 (3)	
H2A	0.647 (4)	0.8074 (10)	0.617 (2)	0.063 (5)*	
H2B	0.610 (4)	0.8842 (10)	0.608 (2)	0.061 (5)*	

### Atomic displacement parameters (Å^2^)

**Table:**

	*U*^11^	*U*^22^	*U*^33^	*U*^12^	*U*^13^	*U*^23^
O1	0.0636 (6)	0.0243 (4)	0.0437 (5)	0.0043 (4)	0.0167 (4)	−0.0011 (3)
C2	0.0426 (6)	0.0264 (6)	0.0346 (6)	0.0016 (4)	0.0092 (5)	−0.0006 (4)
N1	0.0611 (7)	0.0367 (6)	0.0413 (6)	0.0057 (5)	0.0168 (5)	−0.0060 (4)
N2	0.0752 (8)	0.0257 (6)	0.0434 (6)	0.0018 (5)	0.0256 (6)	0.0023 (4)
N3	0.0844 (10)	0.0318 (6)	0.0616 (8)	0.0114 (6)	0.0260 (7)	0.0054 (5)
C4	0.0504 (7)	0.0306 (7)	0.0367 (6)	0.0018 (5)	0.0145 (5)	0.0021 (4)
C3	0.0420 (6)	0.0358 (6)	0.0369 (6)	0.0029 (5)	0.0083 (5)	−0.0038 (5)
C5	0.0622 (8)	0.0581 (9)	0.0384 (7)	0.0072 (7)	0.0180 (6)	0.0027 (6)
C1	0.0440 (6)	0.0237 (6)	0.0369 (6)	−0.0003 (4)	0.0090 (5)	−0.0026 (4)

### Geometric parameters (Å, º)

**Table:**

O1—C1	1.3383 (13)	N2—H2A	0.842 (19)
O1—N1	1.4367 (13)	N2—H2B	0.870 (18)
C2—C1	1.3836 (16)	N3—C4	1.1424 (16)
C2—C4	1.4098 (16)	C3—C5	1.4877 (17)
C2—C3	1.4204 (16)	C5—H5A	0.9600
N1—C3	1.2988 (16)	C5—H5B	0.9600
N2—C1	1.3154 (16)	C5—H5C	0.9600
			
C1—O1—N1	108.74 (8)	C2—C3—C5	127.57 (12)
C1—C2—C4	126.86 (10)	C3—C5—H5A	109.5
C1—C2—C3	104.75 (10)	C3—C5—H5B	109.5
C4—C2—C3	128.25 (11)	H5A—C5—H5B	109.5
C3—N1—O1	105.83 (9)	C3—C5—H5C	109.5
C1—N2—H2A	119.3 (12)	H5A—C5—H5C	109.5
C1—N2—H2B	122.4 (11)	H5B—C5—H5C	109.5
H2A—N2—H2B	116.3 (16)	N2—C1—O1	117.57 (10)
N3—C4—C2	178.79 (15)	N2—C1—C2	133.44 (11)
N1—C3—C2	111.67 (11)	O1—C1—C2	109.00 (10)
N1—C3—C5	120.74 (11)		
			
C1—O1—N1—C3	0.14 (14)	N1—O1—C1—N2	179.10 (11)
O1—N1—C3—C2	0.70 (14)	N1—O1—C1—C2	−0.94 (13)
O1—N1—C3—C5	−178.01 (10)	C4—C2—C1—N2	−2.8 (2)
C1—C2—C3—N1	−1.26 (14)	C3—C2—C1—N2	−178.75 (15)
C4—C2—C3—N1	−177.17 (12)	C4—C2—C1—O1	177.29 (12)
C1—C2—C3—C5	177.35 (12)	C3—C2—C1—O1	1.31 (13)
C4—C2—C3—C5	1.4 (2)		

### Hydrogen-bond geometry (Å, º)

**Table:**

*D*—H···*A*	*D*—H	H···*A*	*D*···*A*	*D*—H···*A*
N2—H2*A*···N1^i^	0.842 (19)	2.118 (19)	2.9567 (16)	174.2 (16)
N2—H2*B*···N3^ii^	0.870 (18)	2.174 (18)	3.0402 (17)	173.8 (15)

Symmetry codes: (i) *x*−1/2, −*y*+3/2, *z*+1/2; (ii) −*x*+1, −*y*+2, −*z*+1.
